# Obesity in pregnancy: a novel concept on the roles of adipokines in uterine contractility

**DOI:** 10.3325/cmj.2017.58.96

**Published:** 2017-04

**Authors:** Judit Hajagos-Tóth, Eszter Ducza, Reza Samavati, Sandor G. Vari, Robert Gaspar

**Affiliations:** 1 Department of Pharmacodynamics and Biopharmacy, University of Szeged, Szeged, Hungary; 2 International Research and Innovation in Medicine Program Cedars - Sinai Medical Center, Los Angeles, CA, USA

## Abstract

Obesity is a global health problem even among pregnant women. Obesity alters quality of labor, such as preterm labor, prolonged labor, and higher oxytocin requirements in pregnant women. The most important factors to play a role in the altered gestational period and serve as drug targets to treat the consequences are female sexual hormones, calcium channels, adrenergic system, oxytocin, and prostaglandins. However, we have limited information about the impact of obesity on the pregnant uterine contractility and gestation time. Adipose tissue, which is the largest endocrine and paracrine organ, especially in obesity, is responsible for the production of adipokines and various cytokines and chemokines, and there are no reliable data available describing the relation between body mass index, glucose intolerance, and adipokines during pregnancy. Recent data suggest that the dysregulation of leptin, adiponectin, and kisspeptin during pregnancy contributes to gestational diabetes mellitus and pre-eclampsia. A preclinical method for obese pregnancy should be developed to clarify the action of adipokines and assess their impact in obesity. The deeper understanding of the adipokines-induced processes in obese pregnancy may be a step closer to the prevention and therapy of preterm delivery or prolonged pregnancy. Gestational weight gain is one of the factors that could influence the prenatal development, birth weight, and adiposity of newborn.

Obesity is a global health problem among pregnant women and an increasing problem in medical practice (1,2). The most negative consequence of obesity is the development of chronic low-grade metabolic inflammation, which can lead to other pathologic conditions (3). Obesity increases the risks of infertility and complications of delivery. In addition, it is not only associated with maternal morbidity, but also causes deleterious health consequences in the offspring (4-6). Obesity may alter the pregnant uterine contractility and hence make the gestation period shorter or longer (7) (Table 1).

**Table 1:** Weeks of preterm, term, and postterm birth (7)

Classification of birth	Gestational period (weeks)
Preterm	22-36
Late preterm	34-36
Term	37-41
Early term	37-38
Full term	39-41
Postterm	42 or greater

Preterm delivery (PD) is childbirth before 37 weeks but after 22 weeks of gestation (8,9). Approximately 15 million babies are preterm worldwide, the rates varying across countries (10). The rate of singleton preterm births seems to decrease slightly and remains below 6% in many European countries (11). However, in Austria, Belgium, Cyprus, Hungary, Germany, or Romania, the preterm birth rate is still close to or slightly beyond 10% (12).

The previous RECOOP HST Association study identified body mass index as one of the most significant factors associated with preterm births in Central and Eastern Europe (13).

Postterm pregnancy (PP), also called late-term and post-term pregnancy, is a gestational period longer than 42 completed weeks from the last menstrual period. The frequency of PPs is highly variable, between 0.5% and 10%, and shows great differences among the countries. Paternal genetics, maternal height, obesity, and male fetal gender are among the factors that may have impact on PP (14). Although the PP incidence shows an increasing tendency, this type of disorder is still depreciated (15).

The exact process of PD is not clear. Decidual hemorrhage, cervical incompetence, uterine distortion, cervical and maternal inflammation, hormonal changes, uteroplacental insufficiency, preeclampsia, multiple gestation, maternal periodontal disease, and fetal disorders are among the factors that may lead to PD. Additional factors are overweight or underweight before or during pregnancy, hypertension, gestational diabetes, blood clotting disorders, in vitro fertilization, extreme maternal age (less than 17 or more than 35 years old), nonwhite race, and poverty (13,16).

The risks factors of PP are not clarified. Recently, the obesity is the only one risk factor of PD that is supposed to be preventable (17). The prevention and treatment of PP is also a challenge in obstetrics because it has many adverse fetal outcomes with either reduced or normal uteroplacental functions (18). German Birth Cohort Studies in newborns and mothers observed statistically significant associations between gestational weight gain (GWG) starting in the second trimester and newborn cord blood and post-delivery maternal serum leptin (19).

## Physiological factors and therapeutic targets of pregnant uterine contraction

### Female sexual hormones

The majority of the mechanisms that lead to delivery are under the control of estrogen and progesterone. Progesterone maintains pregnancy by reducing the resting tone of the pregnant myometrium. The estrogen-to-progesterone ratio determines the contractility of the pregnant myometrium. Estrogen enhances, while progesterone reduces the ability to contract (20). It was proved that the high plasma level of progesterone restores the function of β_2_-adrenergic receptors at the end of pregnancy (21). On the other hand, progesterone has anti-inflammatory effect that may have high physiological and therapeutic importance in the inflammatory approach to PD (22). The sexual hormones influence the expressions of aquaporin 5 proteins and may be potential prognostic factors in the presence of symptoms of threatened premature labor (23). The benefit of the preventive administration of 17-alpha-hydroxyprogesterone caproate-(17-OHPC) in high risk PD was proved by several randomized controlled trials; however, the evidence for better neonatal outcomes are not absolutely convincing (24). Pharmacogenomic studies proved that women with a special single nucleotide polymorphism in the progesterone receptor gene are at increased risk of PD during 17-OHPC therapy (25). The disturbance of the nuclear co-regulatory factor of the progesterone receptor and the subsequent low sensitivity of the receptors was proved in the uterine samples from PP (26).

### Calcium channels

Voltage-dependent L-type calcium channels are crucial in the control of pregnant myometrial contractions. Every alteration in the myometrial contractility has an impact on the function of calcium channels and calcium influx into the myometrial cells. Sexual hormones can alter the isoforms of voltage-dependent L-type calcium channels, which may influence contractility and pharmacological reactivity (27). Calcium channel blockers inhibit calcium re-uptake via these channels and induce smooth muscle relaxation. Nifedipine is one of the most frequently used calcium channel blockers for preterm birth with a better side effect profile as compared with β_2_-adrenergic agonists (28,29).

### Adrenergic system

Alpha-adrenergic receptors are responsible for uterine contraction, while β_2_-adrenergic receptors meditate relaxation in the pregnant myometrium. Different types of α1- and α2-adrenergic receptor blockers have been investigated as potential tocolytics, but none of them have been tried in the clinical practice (30-33). The special adrenergic denervation of the pregnant myometrium also contributes to the changes in contractility (34). β_2_-adrenergic agonists relax the smooth muscle by elevating the intracellular level of cyclic AMP, leading to the phosphorylation of the Ca^2+^ channels. A-kinase anchoring proteins (AKAPs) – protein kinase A (PKA) disruptors – enhance the relaxing effect of β_2_-adrenergic agonists (35). The combination of β_2_-adrenergic agonists with Ca^2+^ channel blockers (36) and phosphodiesterase inhibitors (37) were also investigated in preclinical conditions and beneficial co-actions were found. The effectiveness of the therapy with beta-mimetics influences the genotype of the β_2_-receptor. The first study that investigated the pharmacogenetics of β_2_-adrenergic receptor agonist therapy in the preterm labor found that Arg16 homozygosity improved the efficacy of β_2_-adrenergic receptor agonist-induced tocolysis (38).

### Oxytocin

Oxytocin and oxytocin sensitivity of the pregnant myometrium are equally important in the initiation of both term and non-term deliveries (39). The inhibition of the oxytocin receptor reduces the calcium influx and inhibits the pregnant myometrial contractions (40). Oxytocin antagonists, like atosiban, act both at oxytocin and vasopressin 1A receptors (24). The induction of labor always requires external oxytocin in case of weak myometrial contractions. Oxytocin is routinely used labor induction at 41 weeks and later in case of ripe cervix. In prolonged labor, the administration of oxytocin has importance in reducing the need for cesarean section (41).

### Prostaglandins

Cyclooxygenases (COXs) produce prostaglandins that induce myometrial contractility and cervical ripening and are crucial for the initiation and completion of labor. Prostaglandins E_1_ and E_2_ reduce the dose of oxytocin for labor induction, although prostaglandin E_2_ alone did not show a significant increase in pregnant myometrial activity (42). For this reason, prostaglandin E_1_ analogue, misoprostol, is used for labor induction (43). When cervical conditions are not optimal, the administration of prostaglandin E_2_ is recommended to decrease the oxytocin dose (14). COXs inhibitors, such as indomethacin, can be used for tocolysis, although several fetal side effects should be taken into consideration; therefore, their prolonged use is not recommended (44).

## The impacts of maternal obesity on pregnancy

In contrast to pregnant women of normal weight, insulin resistance is higher in obese pregnant women. During the last trimester, the levels of maternal circulating lipid, triglycerides, low- and high-density lipoproteins, and cholesterol are significantly elevated. Maternal circulating amino acid concentrations are also modified due to the higher protein synthesis for fetal development and placental growth. According to these metabolic changes, lipogenesis is dominant in early gestation and lipolysis in late gestation in women with normal body weight. On the other hand, the gestational weight gain is only accompanied by lipolysis, suggesting the exposure of fetus to increased free fatty acids during fetal development (45-47).

Gestational weight gain can lead to the higher incidence of spontaneous miscarriage and congenital abnormalities, such as neural tube defects or congenital heart failure (47). In later gestation, hypertension, preeclampsia, and gestational diabetes mellitus are clinically observed (48). However, several studies demonstrated that gestational weight gain even can lead to PP (49,50). The subsequent macrosomia is associated with higher incidence of labor induction and cesarean section. However, small for gestational age was also more common in obese pregnancies (5). The effect of maternal obesity may also seem contradictory considering the placental weight. It was found that obesity increases placental weight and hypertrophy and this is positively correlated with birth weight (50,51). Other animal studies, however, showed that gestational high-fat diet reduces prenatal development and alters the placental structure (47). Gestational weight gain is also accompanied by a higher incidence of deep venous thrombophlebitis, hemorrhage after labor, breast-feeding difficulty, postpartum depression, and infections. However, infection is very rare in gestational obesity (5,49). The long-term childhood implications include obesity, type 2 diabetes mellitus, respiratory tract infections, chronic lung disease, bronchial asthma, and chronic obstructive pulmonary disease (52). There are many consequences of maternal obesity during pregnancy (Table 2).

**Table 2:** Complications of maternal obesity*

Maternal complications	Fetal outcomes	Childhood outcomes
PD, PP	stillbirth	obesity
Dystocia	fetal and neonatal death	hypertension
Cesarean section	macrosomia	type 2 diabetes mellitus
Gestational diabetes mellitus	fetal growth restriction	respiratory tract infection
Hypertension	congenital abnormalities	bronchial asthma
Eclampsia		COPD

*Abbreviations: PD – preterm delivery; PP – prolonged pregnancy; COPD – chronic obstructive pulmonary disease-

## Metabolic regulation of human myometrial function during pregnancy

Adipose tissue becomes a very significant endocrine and paracrine organ in obesity. It is responsible for the production of adipokines and various cytokines and chemokines. Adipokines play an important role in hemostasis, lipid degradation, blood pressure control, atherosclerosis, insulin sensitivity, and angiogenesis. In addition, some adipokines are responsible for the immune and inflammation responses (53,54). The potential modulation of contractility by cytokines and inflammatory processes plays a significant role in the changes in pregnancy and gestational age (55). The expanding adipose tissue increases the chronic low-grade inflammation cytokines productions via immune cells. This process induces in metabolic complications (56). The adipose tissue functions are controlled via the brain, since many neuropeptides and neurotrophic factors and their receptors are expressed in both adipose tissue and the central nervous system (53). Since adipokines influence the central and peripheral hypothalamic-pituitary-gonadal axis, adipose tissue is involved in the onset of puberty, the regulation of sexual behavior and fertility, their adaptation to the availability of energy, and the size of fat (3). Leptin, resistin, tumor necrosis factor-alpha, omentin-1, chemerin, ghrelin, visfatin, interleukin-6, kisspeptin, and adiponectin belong to the adipokines (57). Recent data suggest that the dysregulation of leptin, adiponectin, and kisspeptin during pregnancy contributes to diabetes mellitus and pre-eclampsia (54). In addition, obesity was demonstrated to be accompanied by the decreased level of adiponectin and the increased expression of leptin and kisspeptin (58); therefore, there is a growing interest in these adipokines concerning obesity and pregnancy.

### Leptin

Leptin, a 16 kDa protein, plays an important role in the regulation of food intake and energy consumption. It reduces appetite by stimulating the hypothalamic leptin receptor (Ob-Rb) (58); therefore, dysfunction of the hypothalamus or leptin signaling deficiencies may contribute to the development of increased body weight. Leptin was also demonstrated to influence reproductive function (59). The expression of leptin and Ob-Rb mRNAs and proteins was demonstrated in human endometrium and placental trophoblast (60), suggesting the role of leptin in the implantation process. It was also demonstrated that leptin stimulates human trophoblast invasion due to its effect on matrix metalloproteinase (MMP)-2 and MMP-9, which play an important role in implantation (61). The serum concentration of leptin is significantly increased during pregnancy and decreased after birth, revealing an important role during gestation. It also takes part in placental nutrient transfer (62). Two independent German cohort studies provided evidence on the robust association among GWG, fetal-maternal leptin, and fetal development, along with the long-term consequences of hypo- and hyper-leptinemia during pregnancy (19). Leptin was demonstrated to have relaxing effect on isolated human and rat myometria (62,63). The dysregulation of leptin also can lead to infertility. Leptin deficient mice were described to have high rates of insulin resistance and infertility (58).

### Adiponectin

Adiponectin plays an important role in insulin sensitivity and inflammation. It circulates in the blood in high concentrations, as the most abundant adipokine in the circulation. It has an effect on the liver, the skeletal muscle and the vascular system, improves hepatic insulin sensitivity and decreases vascular inflammation (64). Adiponectin acts on different adiponectin receptors. AdipoR1 is localized in the skeletal muscle and stimulates AMPK and leads to lipid oxidation. AdipoR2 can be found in the liver and increasing susceptibility for insulin. The third receptor, t-cadherin (or adiponectin-binding protein) can be found in the endothelium and the smooth muscle (58,64,65). Contrary to leptin, the serum level of adiponectin is inversely correlated with obesity, hypertension, serum lipids and coronary artery disease (66). Decreased adiponectin level is responsible for the development of type 2 diabetes mellitus. The adiponectin level decreases progressively during normal pregnancies, probably in response to reduced insulin sensitivity, and a higher degree of decrease can be detected in pregnant women with obesity or gestational diabetes mellitus (67). Although the signaling pathway of adiponectin (AMPK) has been identified as a mechanism important to the myometrium, no information is available about the adiponectin effect on uterine contractility.

### Kisspeptin

The hypothalamic hormone kisspeptin was first discovered as a metastasis suppressor peptide. It was shown later that it plays an important role in sexual maturation and pubertal development. It binds to the kisspeptin receptor (KISS1R). The deficiency of kisspeptin can lead to idiopathic hypogonadotropic hypogonadism with low level of sexual steroids and gonadotropin hormones, as kisspeptin modifies the secretion of GnRH and increases the level of LH and FSH directly (68-70). It was also demonstrated that there are more kisspeptin neurons in the female than male rodent hypothalamus. Kisspeptin is essential in placental and fetal developments. The level of kisspeptin is elevated during gestation. In addition, high placental expression of KISS1R and kisspeptin has been found in the first trimester (68). Low kisspeptin levels in early pregnancy were found to be accompanied by small gestational weight and recurrent pregnancy loss (71). Obesity alters the expression of KISS1R and kisspeptin regulates glucose homeostasis and can influence the body weight (69,72). Kisspeptin as a neuropeptide is also produced by adipose tissue (73). Mice models of obesity and type 2 diabetes mellitus revealed increased hepatic kisspeptin expression and kisspeptin plasma levels. Human liver samples from type 2 diabetes patients showed increased kisspeptin productions. A growing number of experiments demonstrate that leptin action on GnRH neurons is mediated by kisspeptin. GnRH neurons express the KISSR1, while Ob-Rb is present in kisspeptin neurons in the hypothalamus (3). According to this data, obesity can lead to the overexpression of these adipokines, which can lead to pathophysiological changes.

## Uterine contractility in obese women

According to our recent knowledge, obesity can lead to both premature birth and increased incidence of cesarean section, while weight gain mainly leads to poor myometrial contractility (74). Although there are several studies focusing on the correlation between obesity and the increased prevalence of cesarean section, the exact underlying mechanisms and pathogenesis of the poor myometrial contractility are still questioned.

Increased body weight is positively correlated with increased blood cholesterol level and responsible for changes in myometrial contractility (74,75). Cholesterol is an essential component in the cell membranes. Several components, which are vital in the regulation of smooth muscle signaling pathway, are localized in cholesterol-rich parts of the cell membrane, such as caveolae (76). In animal model, a high-fat high-cholesterol diet decreased the expressions of connexin-43 and caveolin-1 (myometrial contraction-associated proteins), but it increased the expression of COX-2, which can lead the poor myometrial contractility during obesity (77,78). The high-fat high-cholesterol diet was also found to increase the expression of the oxytocin receptor (79), but the existing data are contradictory about the influence of obesity on this receptor (80). The high-fat high-cholesterol diet also resulted in elevated plasma progesterone level at term, which may also give an evidence for the poor contractility during obesity (79). Decreased cervical ripening was also observed during obesity (81,82). Increased leptin in obesity can induce adipose and placental PGE_2_ release contributing to inflammatory processes. It also disrupts collagen degradation and apoptosis. In addition, it promotes cervical collagen synthesis in late gestation, which can also result in delayed labor (50). Leptin also inhibits spontaneous and agonist induced uterine contractions on isolated human and rat myometrium (62,63).

## Conclusions

Gestational weight gain is an important factor during early fetal development and may influence long-term health of newborns. There is an emerging need to clarify the impacts of obesity and adipose tissue-produced adipokines (leptin, adiponectin, kisspeptin) in pregnancy. A preclinical method for obese pregnancy should be worked out to clarify the action of adipokines and assess their impact in obesity. Meanwhile, a clinical investigation should be launched in parallel to reveal the character and the mechanisms of the obesity-induced alteration in gestation length and myometrial contractility. The deeper understanding of the adipokines-induced processes in obese pregnancy may be a step closer to the prevention and therapy of preterm delivery or postterm pregnancy.
